# Immunometabolism of Tregs: mechanisms, adaptability, and therapeutic implications in diseases

**DOI:** 10.3389/fimmu.2025.1536020

**Published:** 2025-01-23

**Authors:** Yuming Lu, Yifan Wang, Tiantian Ruan, Yihan Wang, Linling Ju, Mengya Zhou, Luyin Liu, Dengfu Yao, Min Yao

**Affiliations:** Department of Immunology, Medical School of Nantong University & Research Center of Clinical Medicine, Affiliated Hospital of Nantong University, Nantong, China

**Keywords:** immunometabolism, Tregs, metabolic diseases, metabolic pathways, microenvironment, inflammation

## Abstract

Immunometabolism is an emerging field that explores the intricate interplay between immune cells and metabolism. Regulatory T cells (Tregs), which maintain immune homeostasis in immunometabolism, play crucial regulatory roles. The activation, differentiation, and function of Tregs are influenced by various metabolic pathways, such as the Mammalian targets of rapamycin (mTOR) pathway and glycolysis. Correspondingly, activated Tregs can reciprocally impact these metabolic pathways. Tregs also possess robust adaptive capabilities, thus enabling them to adapt to various microenvironments, including the tumor microenvironment (TME). The complex mechanisms of Tregs in metabolic diseases are intriguing, particularly in conditions like MASLD, where Tregs are significantly upregulated and contribute to fibrosis, while in diabetes, systemic lupus erythematosus (SLE), and rheumatoid arthritis (RA), they show downregulation and reduced anti-inflammatory capacity. These phenomena suggest that the differentiation and function of Tregs are influenced by the metabolic environment, and imbalances in either can lead to the development of metabolic diseases. Thus, moderate differentiation and inhibitory capacity of Tregs are critical for maintaining immune system balance. Given the unique immunoregulatory abilities of Tregs, the development of targeted therapeutic drugs may position them as novel targets in immunotherapy. This could contribute to restoring immune system balance, resolving metabolic dysregulation, and fostering innovation and progress in immunotherapy.

## 1 Introduction

Metabolism, the fundamental process that organisms utilize to sustain life, can be broadly categorized into synthetic metabolism and breakdown metabolism (1). Synthetic metabolism involves the conversion of external nutrients into the organism’s own components, whereas breakdown metabolism refers to the transformation of a portion of the organism into metabolic byproducts, which are subsequently expelled from the body. The equilibrium between these processes is essential for maintaining life, preserving internal environmental stability, and ensuring continuous self-renewal.

The “Warburg effect” describes the metabolic preference of tumor cells, which differs from that of normal cells, which rely on oxidative phosphorylation (OXPHOS) for energy. Even under conditions of ample oxygen, tumor cells opt for the use of glycolytic metabolism instead of OXPHOS to generate energy. Via this metabolic reprogramming, tumor cells gain a growth advantage by utilizing lactate produced from glycolysis. This phenomenon aids in immune evasion and facilitates the proliferation and metastasis of tumor cells (2). This “aerobic glycolysis” phenomenon was later rediscovered in leukocytes, thus gaining increasing attention and evolving into the expansive field of immunometabolism in immunological research. Today, immunometabolism has become a hot research topic, with numerous studies reporting close connections between immune responses and metabolic pathways, such as the impact of various inflammatory factors, including tumor necrosis factor-alpha (TNF-α), on glucose metabolism (3). This series of studies on immunometabolism confirms that the differentiation and function of immune cells are influenced by various microenvironments within the body, thus leading to metabolic reprogramming and altering metabolic pathways in different environments (2, 3).

Regulatory T cells (Tregs), which are a special subset of CD4^+^ T cells, play a vital role in controlling the body’s immune response. Tregs can be classified into natural Tregs (nTregs) derived from thymic development, peripherally induced Tregs (pTregs) generated from initial CD4^+^ T cells in peripheral organs and tissues, and induced Tregs (iTregs) differentiated after external stimulation (4). Tregs from different sources can recognize not only shared antigens but also source-specific antigens, which is reflected in the heterogeneity of T-cell receptors (TCRs) (5). The heterogeneity of TCRs primarily arises from the random recombination of V (variable), D (diversity), and J (joining) gene segments during T-cell development in the thymus. This genetic recombination creates extensive diversity, enabling each T-cell to possess a unique TCR. This process grants T-cells broad antigen recognition capabilities, allowing them to respond to a wide variety of antigens, including both self and foreign antigens. nTregs predominantly recognize self-antigens, contributing to immune tolerance and preventing the development of autoimmune diseases. pTregs primarily recognize harmless foreign antigens, such as microbial and dietary antigens, maintaining peripheral immune homeostasis and preventing unnecessary immune responses to innocuous antigens. iTregs, on the other hand, are generated from conventional T-cells under conditions of infection or inflammation. They are capable of recognizing pathogen-derived antigens and modulating immune responses to prevent excessive inflammation and immune-mediated damage.

The key transcription factor for Tregs is Foxp3, which plays a crucial role in maintaining immune homeostasis (6). Sustained TCR signaling can maintain Foxp3 expression, thus promoting the inhibitory function of Tregs (7). The anti-inflammatory inhibitory capacity of Tregs dates back to the 1970s (8). Treg cells act as metabolic sensors, detecting both local and systemic metabolic signals such as fluctuations in nutrients and changes in peripheral energy sensors. Upon receiving these signals, Treg cells adjust their intracellular metabolism and anti-inflammatory functions to achieve immune tolerance. In different tissues, Treg cells regulate signaling pathways such as mTOR and AMP-activated protein kinase (AMPK) according to nutrient availability, which influences their proliferation and suppressive capabilities. For instance, in adipose tissue, Treg cells adapt to the lipid-rich environment, suppress inflammation, and maintain tissue homeostasis through mechanisms involving FoxP3 and the metabolism of prostaglandin E2, thus regulating local immune responses and preventing excessive inflammation. In the gut, Treg cells are influenced by signals from microbial fermentation products and gut hormones, adjusting their metabolism to maintain mucosal tolerance and epithelial barrier integrity, thereby preventing excessive immune responses that could damage intestinal tissues (9).

This study elucidates the connections between Tregs and relevant metabolic pathways, as well as different metabolic processes. The adaptability of Tregs in diverse microenvironments is also explored. From the perspectives of disease pathogenesis, adaptability, and drug therapy, this article describes the phenotypic differences, dynamic changes, and functional alterations of Tregs in various metabolic diseases. These findings highlight the bidirectional regulatory role of Tregs in disease progression. Finally, the article addresses the topic from a drug therapy standpoint, thus indicating the current efficacy and prospects of therapeutic drugs for different diseases. This study will reveal the diversity of Tregs that are involved in immuno- metabolism, thus emphasizing the critical importance of Tregs stability in immune metabolism.

## 2 The critical signaling or metabolic pathways involved in Tregs

**Figure 1:**
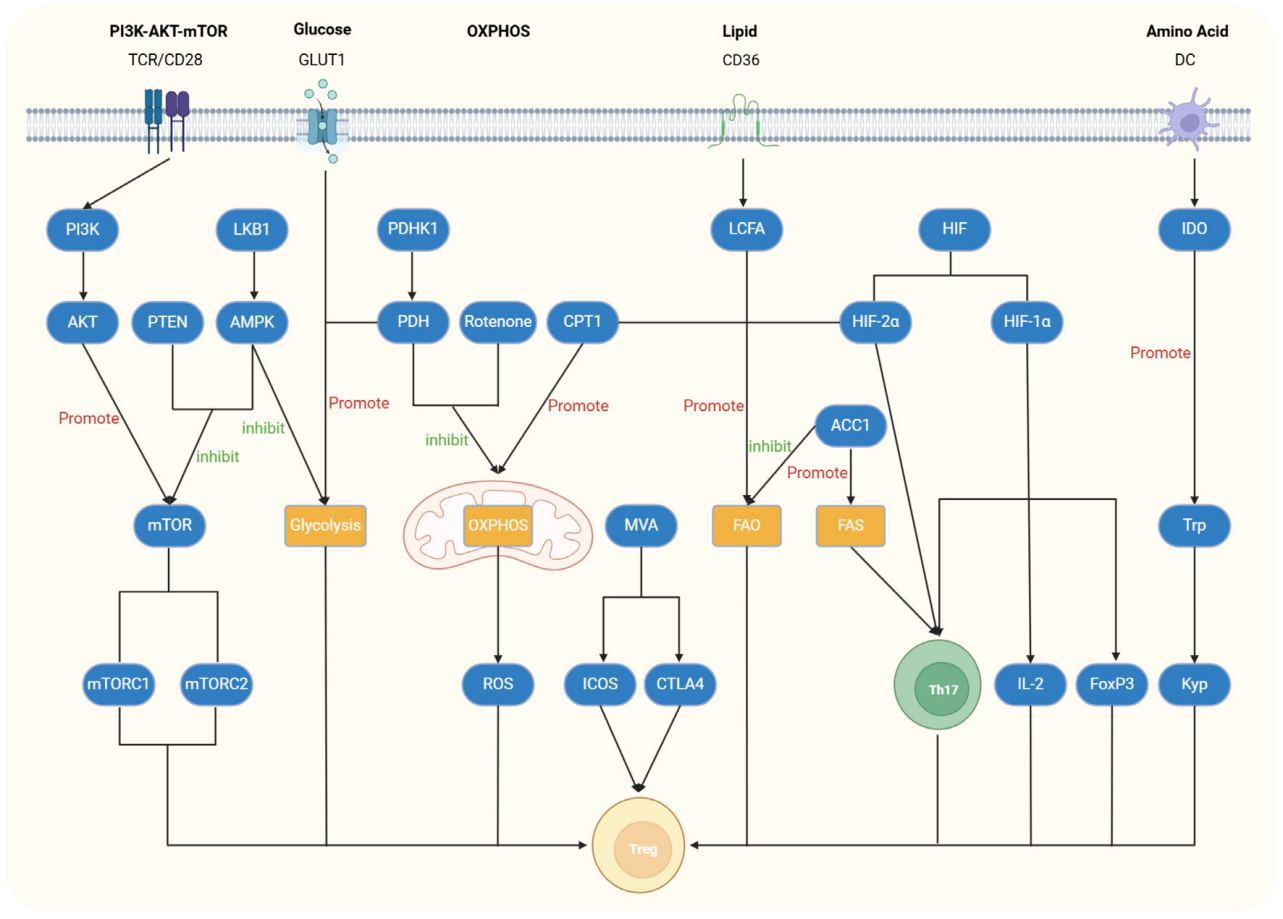
Regulatory role of relevant signaling or metabolic pathways in Tregs. The PI3K-AKT-mTOR pathway is activated by stimulation of T-cell receptors (TCRs) and CD28. The upstream activation of PI3K triggers downstream activation of AKT and Mammalian targets of rapamycin (mTOR), leading to reduced Foxp3 expression and inhibition of Treg cell differentiation and function. Activation of liver kinase B1 (LKB1), AMP-activated protein kinase (AMPK), or Phosphatase and Tensin Homolog (PTEN) can inhibit this pathway and restore Treg differentiation and function. In the glycolytic pathway, glucose transporter 1 (GLUT1) plays a crucial role, and its deficiency inhibits this pathway while enhancing the proliferative capacity of Treg cells. Pyruvate dehydrogenase kinase 1 (PDHK1), as an inhibitor of Pyruvate dehydrogenase (PDH), promotes glycolysis, thereby reducing Treg differentiation and function. Similarly, LKB1, which inhibits the mTOR pathway, also exerts an inhibitory effect in the glycolytic pathway. In the oxidative phosphorylation (OXPHOS) pathway, Carnitine palmitoyltransferase 1 (CPT1) enhances this process, activating acetyl-CoA and increasing reactive oxygen species (ROS) production, which promotes Treg differentiation. Conversely, PDHK1 and mitochondrial electron transport chain inhibitors such as rotenone inhibit OXPHOS, thereby suppressing Treg differentiation and function. In lipid metabolism, Treg cells absorb Long-chain fatty acids (LCFAs) through CD36, promoting the fatty acid oxidation (FAO) pathway and inducing their differentiation. CPT1 and hypoxia-inducible factor-2α (HIF-2α) also participate in the FAO pathway, promoting Treg differentiation. Acetyl-coenzyme A carboxylase 1 (ACC1) not only promotes the fatty acid synthesis (FAS) pathway and Th17 cell differentiation but also inhibits FAO and Treg differentiation. In amino acid metabolism, Indoleamine 2,3-dioxygenase (IDO) converts tryptophan into kynurenine, thereby promoting the proliferation, differentiation, and function of Treg cells.

The development, differentiation, and functional maintenance of Tregs are intricately regulated by key signaling pathways and metabolic processes. Their activation and proliferative capacity primarily depend on pathways such as the mTOR pathway and glycolysis, whereas their functionality relies heavily on lipid metabolism and other metabolic pathways. Herein, we provide a summary of the crucial signaling pathways and various metabolic processes that play regulatory roles in the differentiation, function, and homeostasis of Tregs (**Figure 1**).

### 2.1 PI3K-AKT-mTOR pathway

The T cell receptor (TCR) can activate the PI3K-AKT-mTOR signaling pathway, in which phosphoinositide 3-kinase (PI3K) is activated by the TCR and subsequently activates downstream AKT. Phosphorylated AKT can inhibit the expression of Foxp3, thereby suppressing the differentiation and function of Tregs (10). PI3K, which is positioned upstream of mTOR, contributes to the differentiation and function of Tregs when weakened. mTOR, which is a noncanonical serine/threonine protein kinase, exists in two complexes (mTORC1 and mTORC2) (11). mTOR plays crucial regulatory roles in the activation, proliferation, and function of Tregs (12). Specifically, mTOR regulates the availability of nutrients, particularly glucose and lipid metabolism, influencing the differentiation and function of Tregs. mTORC1 senses the cellular nutrient status (e.g., amino acids, glucose) to regulate Treg metabolism, promoting their proliferation and immune suppressive function. In glucose metabolism, mTORC1 promotes glycolysis, which is crucial for Treg activation and function. Simultaneously, mTORC1 also influences lipid metabolism, regulating the synthesis and breakdown of fatty acids, thus supporting Treg proliferation and maintenance.

Studies have indicated that Tregs lacking Phosphatase and Tensin Homolog (PTEN) enhance mTOR and impair Tregs differentiation and function, thereby promoting the development of various metabolic diseases (13). The serine-threonine kinase liver kinase B1 (LKB1) activates AMPK, thus inhibiting glucose transporter 1 (GLUT1) (This membrane protein is responsible for transporting glucose from the extracellular space into the cell. It belongs to the glucose transporter (GLUT) family and is widely expressed in various tissues, especially in cell types that require a continuous supply of glucose, such as the brain, red blood cells, and embryonic tissues) and mTOR and promoting Tregs differentiation and function (14, 15). Tregs lacking LKB1 exhibit compromised anti-inflammatory functions (16).

### 2.2 Glycolytic metabolism and hypoxia

Compared with those in helper CD4^+^ T cells, GLUT1 expression levels are lower in Tregs. Therefore, GLUT1 deficiency or the use of glycolysis-inhibiting drugs such as 2-DG (2-deoxy-D-glucose) can increase the proliferation and viability of Tregs (17).

There is considerable controversy regarding the impact of hypoxia on Tregs. Some studies suggest that hypoxia-inducible factor-1α (HIF-1α) is upregulated during inflammation, thus increasing the expression of IL-2 and Foxp3 and thereby promoting the activation and proliferation of Tregs (18, 19). However, many other studies have reported that HIF-1α promotes the generation of Th17 cells and that its binding to Foxp3 leads to its degradation, thereby inhibiting the differentiation and function of Tregs (20–22).

Additionally, hypoxia-inducible factor-2α (HIF-2α) has been reported to influence the differentiation and function of Tregs, thus exhibiting a bidirectional regulatory effect. HIF-2α can restore the inhibitory function of Tregs by inhibiting HIF-1α, which reduces the differentiation of Th17 cells. It can also indirectly enhance glycolysis by inhibiting fatty acid oxidation (FAO), thereby suppressing the differentiation and function of Tregs (23). Furthermore, the coactivator of the Nedd4 family of E3 ubiquitin ligases (Ndfip1) maintains the stability of Tregs by limiting mTORC1 signaling and glycolysis. Although the number of Tregs lacking Ndfip1 remains unchanged, their phenotype undergoes significant alterations. These cells exhibited a highly proliferative state, elevated IL-4 levels, and a tendency to lose Foxp3 expression. This metabolic adaptation might be linked to the onset of inflammation (24).

### 2.3 Oxidative phosphorylation metabolism

FOXP3 can upregulate the activity of all the mitochondrial respiratory complexes, thus promoting OXPHOS and FAO (25). Unlike other T cells, Tregs can maintain their differentiation status and activity through the OXPHOS pathway. Consequently, Tregs can utilize OXPHOS to compensate for losses incurred by other impaired metabolic pathways, thus ensuring the continued maintenance of their differentiation and function. However, excessive enhancement of OXPHOS in Tregs may increase mitochondrial reactive oxygen species (ROS) production, thus leading to disruptions in cellular redox homeostasis and oxidative stress (26).

Carnitine palmitoyltransferase 1 (CPT1) enhances OXPHOS, activates acetyl- coenzyme A, and increases the number of Tregs (27). Conversely, mitochondrial electron transport chain inhibitors such as rotenone can inhibit mitochondrial OXPHOS, thus impairing the suppressive ability of Tregs (28). Pyruvate dehydrogenase (PDH) converts pyruvate to acetyl-coenzyme A, thus promoting mitochondrial OXPHOS. Pyruvate dehydrogenase kinase 1 (PDHK1), which is highly expressed in Th17 cells, inhibits PDH, which promotes aerobic glycolysis while inhibiting OXPHOS, thereby reducing the differentiation and function of Tregs. The inhibition of PDHK1 can weaken Th17 cells, enhance Tregs, and reduce the occurrence and development of inflammation (29).

### 2.4 Lipid metabolism

Tregs absorb long-chain fatty acids through CD36, thus enabling their stable participation in the FAO pathway (30). Tregs can regulate the activity of CPT1, which leads to an increase in FAO. Inhibitors of CPT1 suppress Tregs differentiation, thus impacting FAO (31). Additionally, the upregulation of fatty acid synthesis (FAS) promotes the differentiation of Th17 cells, which downregulates FAO. Due to the fact that FAO is essential for Tregs differentiation, lipid metabolism to some extent determines the differentiation fate of CD4^+^ T cells. The inhibition of both FAS and FAO results in an imbalance between Th17 cells and Tregs (32). In a highly glycolytic environment, Th17 cells promote *de novo* fatty acid synthesis via acetyl-coenzyme A carboxylase 1 (ACC1). The inhibition of ACC1 not only suppresses glycolytic metabolism and *de novo* fatty acid synthesis but also reduces Th17 cell differentiation while increasing Tregs production (33). Methylhydroxybutyrate is crucial for the proliferation and inhibitory function of Tregs. The inhibition of the synthesis of methylhydroxybutyrate reduces the expression of inducible costimulatory molecule (ICOS) and cytotoxic T-lymphocyte-associated protein 4 (CTLA4), thus weakening the inhibitory capacity of Tregs and enhancing inflammation (34). Moreover, the basic leucine zipper ATF-like transcription factor (BATF) can inhibit triglyceride synthesis. Tregs lacking BATF exhibit a significant increase in triglyceride expression, which severely affects the differentiation and function of Tregs (35).

### 2.5 Amino acid metabolism

Indoleamine 2,3-dioxygenase (IDO) in dendritic cells (DCs) and tumor cells converts tryptophan into kynurenine, thus promoting the proliferation and differentiation of Tregs (36). The absence of glutamine can inhibit mTOR, thus leading to biased differentiation of Th1 cells toward Tregs (37). Studies have shown that the small G proteins Rag and Rheb maintain mTOR activity by regulating amino acid metabolism, thereby inhibiting the differentiation and function of Tregs (38).

## 3 Adaptability of Tregs in different microenvironments

**Figure 2:**
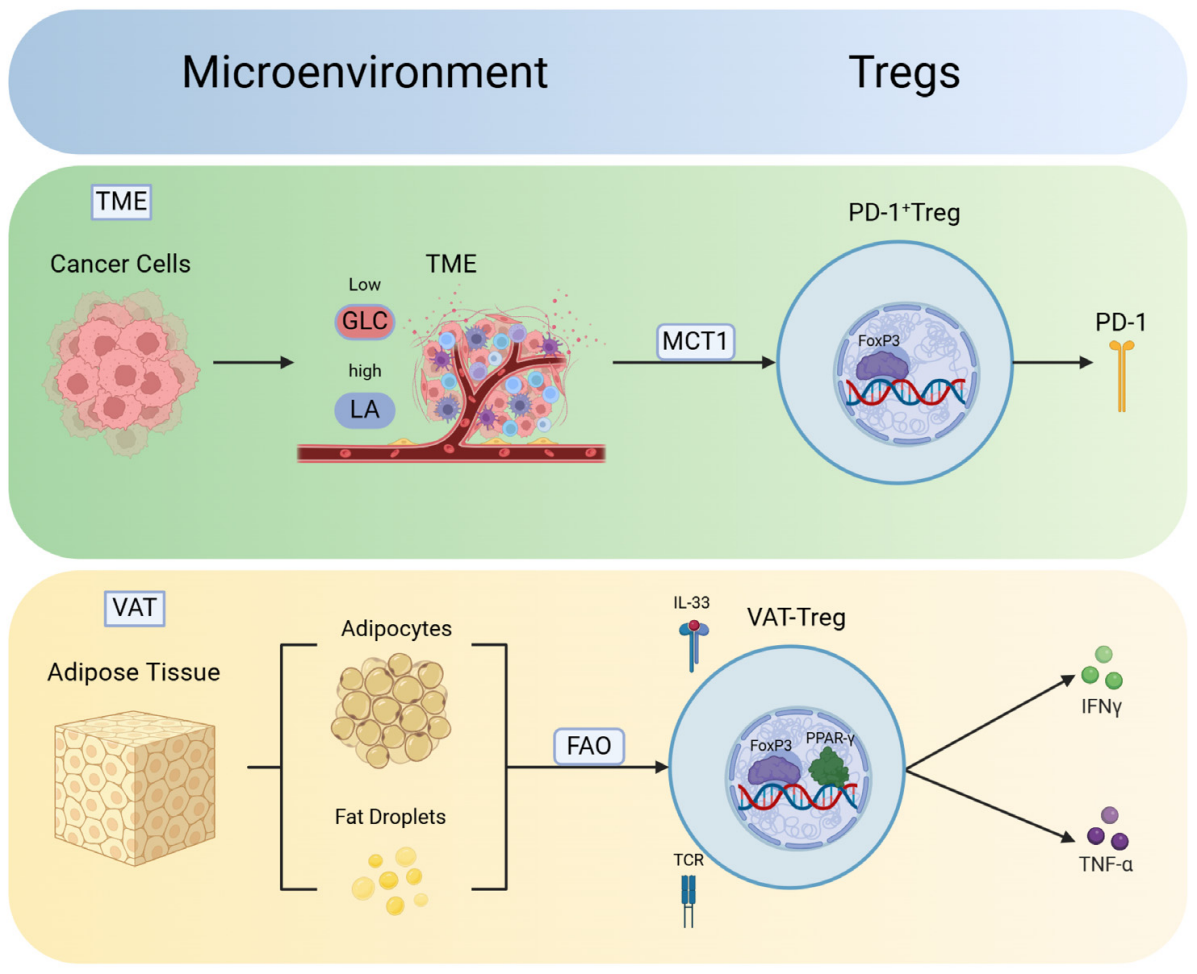
Phenotype and function of Tregs vary in different microenvironments. Tregs exhibit robust adaptability in different microenvironments, thus leading to the development of unique phenotypes. These findings indicate the plasticity of Tregs in response to various environmental conditions. Moreover, Tregs in different microenvironments acquire distinct functions to adapt to changes in the environment and phenotype. In the tumor microenvironment (TME), Tregs absorb lactate (LA) via monocarboxylate transporter 1 (MCT1), thus promoting the generation of PD-1 and differentiation into PD-1^+^ Tregs. In the visceral adipose tissue (VAT), Tregs utilize FAO pathways to mitigate lipotoxicity. T-cell receptors (TCRs) signaling and IL-33 contribute to the expression of Peroxisome proliferator-activated receptor gamma (PPARγ), thus facilitating the differentiation of VAT-Tregs and the release of inflammatory factors such as IFN-γ and TNF-α.

The self-adaptive ability of Tregs allows them to survive in various microenvironments, such as the gastrointestinal tract, liver, and adipose tissue. They undergo metabolic reprogramming based on the specific environment, thus correspondingly altering their phenotype and function. The roles played by Treg cells in different microenvironments can significantly vary. They can play protective anti-inflammatory roles; however, they can also act as pathogenic proinflammatory agents. The generation of these subtypes is often driven by the need to adapt to the specific microenvironment in which Treg cells find themselves (39). In this context, we discuss the adaptability of Tregs in different microenvironments and the resulting changes in their phenotype and function (**Figure 2**).

### 3.1 Tumor microenvironment

Tumor cells compete for nutrient resources from effector T cells, thus converting them into energy and auxiliary factors and creating a tumor-infiltrating microenvironment (TME) (40, 41). Cancer cells in the TME primarily produce lactate via glycolysis, which aids in immune escape. Various cell types, including Tregs, exist in the TME, which consists of tumor cells, immune cells, and fibroblasts, among other cell types (42). The low-glucose, high-lactate TME created by tumor cells not only is harmless to the normal survival of Tregs but also promotes their proliferation and differentiation (25). Tregs take up lactate (LA) through monocarboxylate transporter 1 (MCT1) and increases the expression of PD-1, thus activating the PD-1^+^ Treg subset. This subset significantly inhibits the function of effector T cells, such as CD8^+^ T cells, thus rendering anti-PD-1 therapy ineffective (43).

### 3.2 Visceral adipose tissue

Visceral adipose tissue (VAT) serves as the primary site for energy storage in organisms, whereby it regulates biological activities through the intake of nutrients. VAT-Tregs can adapt to the lipid-rich environment and utilize fatty acids via pathways such as FAO. This adaptation allows them to avoid lipid toxicity while maintaining homeostasis in the VAT environment. However, in obese individuals, the production of type I interferons (IFN-α) secreted by plasmacytoid dendritic cells (pDCs) is increased, which directly inhibits the accumulation of VAT-Tregs, leading to their dysfunction and impaired adaptability in the lipid-rich environment. The quantity of these cells decreases with increases in indicators such as body mass index, thus leading to elevated levels of inflammatory factors such as IFN-γ and TNF-α (44, 45). Peroxisome proliferator-activated receptor gamma (PPARγ) is a key transcription factor that drives the expression of VAT-Tregs. The specific loss of PPARγ significantly reduces the quantity of VAT-Tregs (46). Studies have indicated that TCR signal transduction and IL-33 can increase the number of VAT-Tregs and increase PPARγ expression, thereby reducing inflammation in obese individuals (47).

### 3.3 Central nervous system microenvironment

The central nervous system (CNS) has long been considered an immune-privileged site, but recent evidence indicates that immune cells, including Tregs, can infiltrate the CNS under both physiological and pathological conditions (48). Tregs play a critical role in regulating neuroinflammation, particularly in diseases such as multiple sclerosis (MS) (49). In the CNS microenvironment, Tregs primarily regulate immune responses by inhibiting the activation of microglia and effector T cells. Additionally, they maintain the integrity of the blood-brain barrier, preventing immune cell infiltration and reducing inflammation (50). CNS-resident Tregs undergo metabolic reprogramming via glycolysis and fatty acid oxidation (FAO) to adapt to the nutrient-scarce environment. Moreover, CNS-resident Tregs respond to signals such as IL-10 and TGF-β, regulating neuroinflammatory responses and promoting tissue repair through interactions with glial cells (51).

### 3.4 Respiratory system microenvironment

In the respiratory system, Tregs play a crucial role in maintaining immune homeostasis and preventing excessive immune responses to inhaled antigens, such as allergens, pathogens, and pollutants. In the pulmonary microenvironment, Tregs control inflammation by regulating the activity of alveolar macrophages, dendritic cells, and other effector immune cells (52). They are particularly important in chronic respiratory diseases, where Tregs prevent excessive immune responses and tissue damage (53). Pulmonary Tregs are regulated by signals such as IL-10 and TGF-β, and they undergo metabolic adaptation to efficiently function in the hypoxic and inflammatory environment of the lungs. Furthermore, in cases of acute respiratory infections, such as influenza or COVID-19 viral infections, Tregs help attenuate excessive immune responses, thereby protecting lung tissue from damage caused by inflammation (54).

## 4 Immunometabolism of Tregs in various diseases

**Figure 3:**
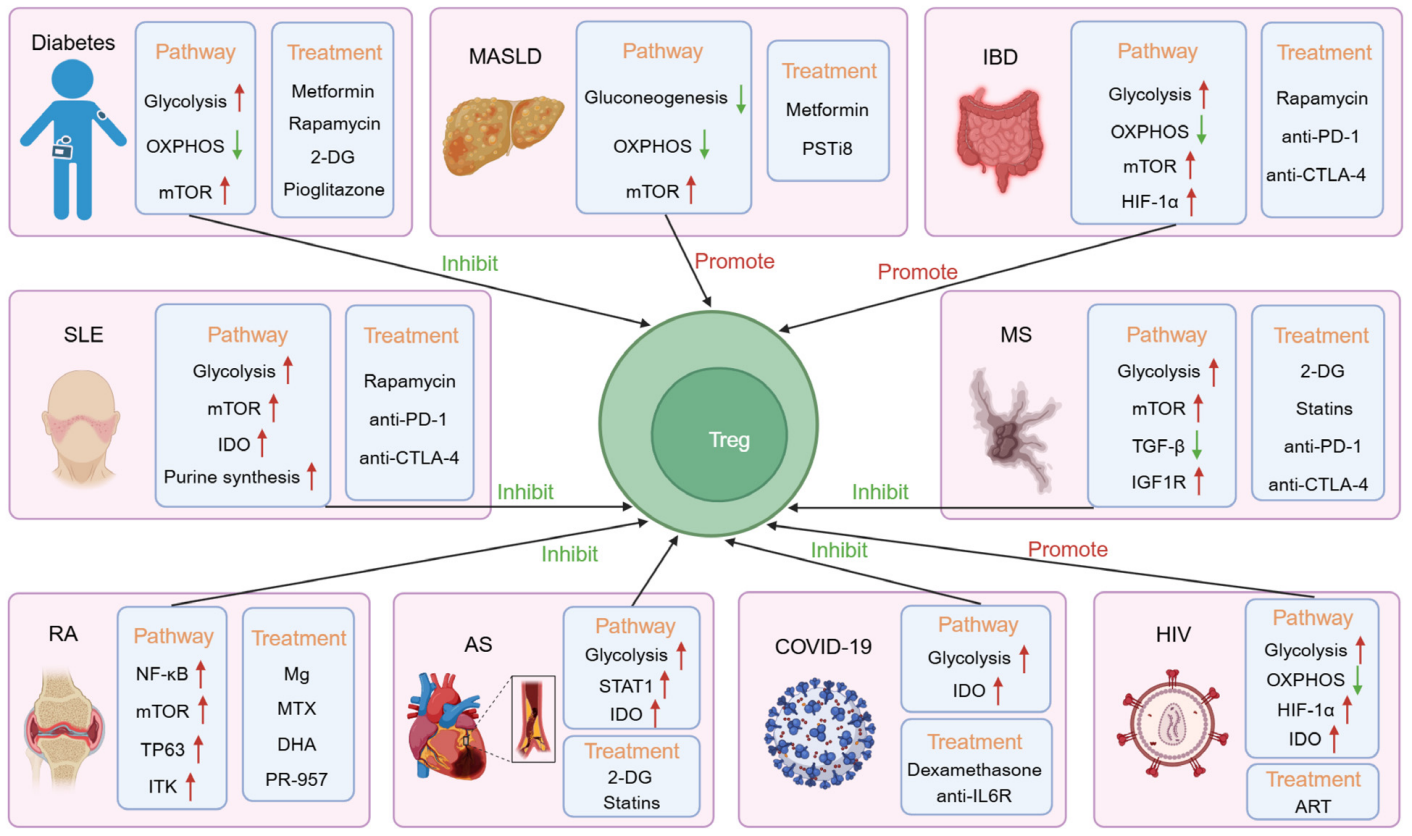
The mechanisms, adaptability, and therapeutic implications of Treg immunemetabolism in various diseases. In diabetes, both glycolysis and the mammalian targets of rapamycin (mTOR) pathway are upregulated, while the oxidative phosphorylation (OXPHOS) pathway is downregulated, which has an inhibitory effect on Tregs. The use of metformin, rapamycin, 2-deoxy-D-glucose (2-DG), and pioglitazone can alleviate the condition. In Metabolic Dysfunction-Associated Steatotic Liver Disease (MASLD), the mTOR pathway is upregulated, while gluconeogenesis and the oxidative phosphorylation pathway are downregulated, promoting Treg function. Metformin and pancreatic suppressor (PST) inhibitor (PSTi8) can alleviate the condition. In Inflammatory Bowel Disease (IBD), glycolysis, mTOR, and the hypoxia-inducible factor-1α (HIF-1α) pathway are upregulated, while the oxidative phosphorylation pathway is downregulated, promoting Treg function. Rapamycin, anti-PD-1, and anti-CTLA-4 can alleviate the condition. In Systemic Lupus Erythematosus (SLE), glycolysis, mTOR, Indoleamine 2,3-dioxygenase (IDO), and purine synthesis pathways are upregulated, inhibiting Treg function. Rapamycin, anti-PD-1, and anti-CTLA-4 can alleviate the condition. In Rheumatoid arthritis (RA), the NF-κB, mTOR, TP63, and interleukin-2-inducible T cell kinase (ITK) pathways are upregulated, inhibiting Treg function. Mg, Methotrexate (MTX), dihydroartemisinin (DHA), and PR-957 can alleviate the condition. In Multiple Sclerosis (MS), glycolysis, mTOR, and Insulin-like Growth Factor 1 Receptor (IGF1R) pathways are upregulated, while the TGF-β pathway is downregulated, inhibiting Treg function. 2-DG, statins, anti-PD-1, and anti-CTLA-4 can alleviate the condition. In Arteriosclerosis (AS), glycolysis, STAT1, and IDO pathways are upregulated, inhibiting Treg function. 2-DG and statins can alleviate the condition. In COVID-19, glycolysis and IDO pathways are upregulated, inhibiting Treg function. Dexamethasone and anti-IL-6R can alleviate the condition. In HIV, glycolysis, HIF-1α, and IDO pathways are upregulated, while the oxidative phosphorylation pathway is downregulated, promoting Treg function. Antiretroviral therapy (ART) can alleviate the condition.

The development of metabolic diseases is significantly influenced by disruptions in Treg metabolism. Metabolic changes, such as altered nutrient availability and energy metabolism, can impact Treg differentiation, function, and stability. For example, shifts in fatty acid oxidation, glycolysis, and mitochondrial function can impair Treg activity, leading to an imbalance in immune regulation. This dysfunction of Tregs may result in either excessive or insufficient immune tolerance, promoting the imbalance of Th17/Treg cells and triggering inflammation, which contributes to the progression of metabolic diseases (55) (**Figure 3**, **Table 1**).

**Table 1:** Differentiation, functions, and therapeutic approaches of tregs in various metabolic diseases.

Disease	Tregs counts	Tregs functions	Treatment elements and drugs
Diabetes (T1D and T2D)	↓	Inhibit the production of CD4 and CD8 T cells, as well as inflammatory cytokines IFN-γ and TNF-α, thus leading to the death of pancreatic β cells; absorb lipids to maintain immune homeostasis and metabolism	Metformin, Rapamycin, Pioglitazone
Metabolic dysfunction-associated steatotic liver disease (MASLD)	↑	Inhibit effector T cells, macrophages and other cells; enhance anti-inflammatory ability; interaction with NETs weakens precancerous immune surveillance	PSTi8
Inflammatory Bowel Disease (IBD) and Colorectal Cancer (CRC)	↑	Some Tregs undergo a transition from an anti-inflammatory state to a pro-inflammatory state; weaken the response of Th1 and CD8 T cells, which are effector T cells involved in anti-tumor responses, and produce inflammatory factors such as IL-10 and TGF-β	Rapamycin, anti-CTLA-4, anti-PD-1
Systemic Lupus Erythematosus (SLE)	↓	Inducing FAO by upregulating CPT1, thereby consuming fatty acids and inhibiting the differentiation of Th17 cells	Metformin, 2-DG, KN-93, Rapamycin, Sirolimus, Pioglitazone, Statins, NAC
Rheumatoid Arthritis (RA)	↓	Produce anti-inflammatory factor IL-10; inhibit the production of pro-inflammatory factors such as TNF-α, IL-1β and IL-6, as well as the activation of NF-κB; reduce pathogenic Th17 cells; alleviate inflammation; reduce joint damage	Mg, MTX, DHA, PR-957, Smad7, JPH203
Multiple Sclerosis (MS)	↓	Inhibit the production of effector T cells and inflammatory factors such as IFN-γ, IL-6 and IL-23 in the central nervous system; do not inhibit the production of IL-17	2-DG, Statins
Atherosclerosis (AS)	↓	Secrete IL-10 and TGF-β; inhibit IFN-γ and other inflammatory factors secreted by macrophages; downregulate scavenger receptor; reduce cholesterol absorption; inhibit DC via CTLA-4	Statins, ApoA1, Arginine
COVID-19	↓	Increase PD-L1 expression	Anti-TNF-α, Anti-IFN-γ, Anti-IL-6, Dexamethasone
AIDS (HIV)	↑	Weaken the immunosuppressive activity of HIV-specific CD8 T cells and their ability to inhibit HIV replication via cAMP	ART

↓: decrease.

↑: increase.

### 4.1 Diabetes

Diabetes is a chronic metabolic disorder characterized by elevated blood sugar levels; in severe cases, it can lead to a range of complications, such as cardiovascular diseases and kidney disorders. It can be classified into type 1 diabetes (T1D) and type 2 diabetes (T2D) (56, 57). T1D is a T cell-mediated chronic metabolic disease characterized by impaired pancreatic beta-cell function (56, 58). Various factors, including genetic and environmental factors, contribute to the development of T1D (59). CD4^+^ and CD8^+^ T cells release inflammatory cytokines such as TNF-α and IFN-γ, thus causing the death of pancreatic beta cells and triggering T1D (60). iTregs play a crucial role in immune tolerance by suppressing the production of inflammatory cytokines, such as IFN-γ, by CD4^+^ and CD8^+^ T cells, and by blocking the mTOR signaling pathway. This helps alleviate inflammation and reduce the severity of T1D (61).

Compared with T1D, T2D accounts for the majority of diabetes cases. T2D is a chronic inflammatory disorder caused by metabolic disturbances in glucose and lipid metabolism and is characterized by insulin resistance and dysfunction of pancreatic beta cells (57, 62). T2D patients exhibit an increased Th1/Treg and Th17/Treg ratio, characterized by a significant elevation in Th1 and Th17 levels and a notable reduction in Treg levels. As a result, there is an increase in pro-inflammatory factors such as IL-6 and IL-17, leading to the formation of a pro-inflammatory environment, while anti-inflammatory factors such as IL-10 are suppressed (63). Tregs are crucial for T2D patients and are capable of absorbing lipids and maintaining immune homeostasis and metabolism. However, T2D patients exhibit a significant reduction in pTregs in adipose tissue, which may be a crucial factor in the development of this disease (44, 64).

The first-line diabetes treatment known as metformin inhibits Th1 and Th17 cell differentiation while enhancing Treg cell proliferation and differentiation, which slows disease progression (65). The mTOR inhibitor rapamycin, combined with IL-2 therapy, can suppress Th17 cells, increase Treg cells, and restore beta-cell function, thus achieving therapeutic effects (66). The glycolysis inhibitor 2-DG balances the Th17/Treg ratio by reducing glycolysis and promoting OXPHOS, thereby increasing the granularity of pancreatic β-cells (67). Additionally, PPARγ-driven lipid metabolism regulates the phenotype of adipose tissue pTregs. The use of synthetic PPARγ agonists such as pioglitazone effectively enhances the expression of lipid metabolism genes, thus allowing pTregs to utilize lipid metabolism for energy and thereby resisting inflammation and reducing the progression of diabetes (44, 68).

### 4.2 Metabolic dysfunction-associated steatotic liver disease

Non-alcoholic fatty liver disease (NAFLD), has been renamed as MASLD, refers to a clinical and pathological syndrome characterized by excessive fat deposition in the liver, with the exclusion of alcohol-related factors. It encompasses a spectrum of conditions ranging from simple hepatic steatosis (SFL) and metabolic dysfunction- associated steatohepatitis (MASH) to liver cirrhosis (LC) and hepatocellular carcinoma (HCC). This progression involves liver fat degeneration, inflammation, hepatocellular damage, and liver fibrosis (69, 70).

The role of Tregs in MASLD is currently a subject of considerable debate. Some studies have suggested that iTregs reduce liver inflammation by suppressing effector T cells, neutrophils, and macrophages (71, 72). It has been reported that a pancreatic suppressor (PST) inhibitor (PSTi8) can treat MASH in mice fed a methionine-choline-deficient (MCD) diet (73). PST regulates glucose and lipid metabolism in the liver and adipose tissue, thus promoting fat degeneration and inflammation. It acts against insulin while altering mitochondrial function (74). PSTi8 enhances Tregs differentiation and strengthens their suppressive ability. Moreover, PSTi8 alleviates hepatic fat degeneration and fibrosis in MCD-fed mice, reduces liver damage markers such as ALT, LDL, and TC, and ameliorates mitochondrial dysfunction. Research has demonstrated that PSTi8 alleviates MASH-related inflammation and fat degeneration by enhancing the anti-inflammatory ability of iTregs. However, at times, the suppressive capabilities of Tregs can be exploited by other diseases and cells, thus leading to unintended consequences. For example, the interaction between Tregs and neutrophil extracellular traps (NETs) may promote the transition from MASH to HCC.

Neutrophils, which are abundant in MASH patients, release chromatin-related traps known as NETs (75). NETs can upregulate the expression of genes related to OXPHOS, increase mitochondrial respiration, and enhance the activity of transcription factors associated with Treg differentiation, thereby increasing the number and function of Tregs. In liver cancer tissues, the increased number of Tregs can suppress immune surveillance through multiple mechanisms, such as inhibiting the function and proliferation of effector T cells (Teffs) and reducing the cytotoxic activity of immune cells against tumor cells. Through Toll-like receptor 4 (TLR4), NETs induce metabolic reprogramming and promote the initial differentiation of CD4^+^ T cells into Tregs via the mitochondrial OXPHOS pathway. This effect weakens immune surveillance in the precancerous stage, thus creating an immune-suppressive environment that is conducive to cancer cell survival and growth (76). Additionally, the dual-regulatory protein of Tregs known as amphiregulin (AREG) can activate hepatic stellate cells (HSCs) via the EGFR signaling pathway, thereby promoting liver fibrosis and insulin resistance in MASH (77).

### 4.3 Inflammatory bowel disease and colorectal cancer

Inflammatory bowel disease (IBD), which is comprised of Crohn’s disease (CD) and ulcerative colitis (UC), is characterized by chronic inflammation in the gastrointestinal tract, which often leads to tissue damage (78). Prolonged IBD can increase the risk of colorectal cancer (CRC) development (79). In general, Tregs play a role in anti-inflammatory responses and the maintenance of immune homeostasis. However, research suggests that in IBD, the upregulation of IL-21 disrupts multiple metabolic pathways, including oxidative phosphorylation (OXPHOS) and glycolysis, thus leading to disturbances in the structure of the mitochondria-endoplasmic reticulum (ER) network (80). This upregulation enhances the activity of glycogen synthase kinase 3β (GSK3β), thus inhibiting the voltage-dependent anion channel (VDAC) in the mitochondria and causing some Tregs to undergo a transition from an anti-inflammatory state to a proinflammatory state. Research has also confirmed that the inhibition of IL-21 or GSK3β can alleviate the symptoms of IBD (81). Additionally, the increase in glycolysis in IBD contributes to the differentiation of Th17 cells, which leads to an imbalance between proinflammatory Th17 cells and anti-inflammatory iTregs, thus further exacerbating inflammation (82).

Numerous studies have indicated that inhibition of the mechanistic target of rapamycin (mTOR) can reduce the differentiation of Th17 cells while increasing the population of anti-inflammatory iTregs (83, 84). These findings suggest that the targeting of mTOR may be a potential therapeutic approach to ameliorate inflammation caused by IBD (83, 84). Moreover, HIF-1α plays a crucial role in controlling the differentiation of Tregs in IBD, and its regulation is vital for improving inflammation (20, 85). Therefore, the targeting of key metabolic hubs (such as mTOR and HIF-1α) to restore the function of anti-inflammatory iTregs could be beneficial for targeted immuno- metabolic therapy in IBD (86).

Furthermore, previous studies have indicated that when IBD progresses to CRC, the number and function of Tregs change, thus resulting in characteristics similar to those of Th17 cells (87). In CRC, tumor cells rely on glycolysis, glutaminolysis, and FAS for metabolic reprogramming. Tregs adapt to these changes in metabolic pathways and enhance their differentiation and inhibitory functions in the TME. This correspondingly weakens the antitumor immune response of Th1 and CD8^+^ T cells, thus promoting the immune escape of tumor cells and contributing to the development of CRC. In CRC, Tregs also express coinhibitory receptors such as LAG-3 and TIM-3 (88). LAG-3^+^ Treg cells increase the production of IL-10 and TGF-β, as well as the expression of CTLA-4. Currently, therapeutic approaches targeting tumor-associated Treg cells, such as anti-CTLA-4 and anti-PD-1 treatments, are crucial for alleviating and suppressing CRC. The combination of anti-CTLA-4 monoclonal antibodies with anti-PD-1 monoclonal antibodies holds promise for achieving enhanced therapeutic effects (89, 90).

### 4.4 Systemic lupus erythematosus

Systemic lupus erythematosus (SLE) is an autoimmune disease characterized by chronic inflammation and excessive activation of immune cells such as B cells and T cells (91). The activation and differentiation of immune cells in SLE patients are closely linked to alterations in metabolic pathways. Metabolic abnormalities can significantly impact immune cell function, thereby exacerbating autoimmune responses and inflammation. For example, in SLE patients, T cells and B cells undergo metabolic reprogramming, which alters their energy metabolism and functional characteristics. This may promote the release of inflammatory cytokines and cause tissue damage. Tregs play a crucial role in these metabolic changes. nTregs suppress inflammation by maintaining immune tolerance, but their function is also affected by metabolic abnormalities, which may lead to immune dysregulation, further exacerbating the pathogenesis of SLE (92, 93).

In SLE mice, oral administration of metformin can increase nTregs, reduce Th17 cells, inhibit mTOR and signal transducer and activator of transcription 3 (STAT3) signaling, as well as decrease the severity of inflammation. Furthermore, the addition of 2-DG can restore T cell metabolism and alleviate inflammation in SLE patients (94, 95). The overexpression of calcium/calmodulin-dependent protein kinase IV (CaMK4) can increase mTOR activity, thus promoting Th17 cell differentiation (96). The CaMK4 inhibitor KN-93 can increase the number of Tregs in diseased mice and decrease the number of inflammatory immune cells, such as Th17 cells (97). Similarly, rapamycin and sirolimus, as mTOR inhibitors, can increase the number of Tregs by inhibiting mTOR, thus reducing pathogenic T-cell subgroups (including Th17 cells) and decreasing proinflammatory cytokines, among others (98, 99). Additionally, the insulin sensitizer pioglitazone can increase the proportion of Tregs by inhibiting mTOR and activating AMPK in SLE patients (100).

Acetyl-CoA carboxylase 1 (ACC1) in lipid metabolism is an important regulator of Th1 and Th17 cells, thus promoting their differentiation via metabolic reprogramming (33, 101). Conversely, Tregs induce FAO by upregulating CPT I, thus inhibiting the differentiation of Th17 cells by consuming fatty acids. Statin drugs may also help in regulating the balance between Tregs and Th17 cells, thus contributing to the treatment of SLE patients (102). In amino acid metabolism, the expression of IDO can limit the secretion of inflammatory cytokines, promote the generation of Treg cells, and regulate the Th17/Treg balance (103). Additionally, mycophenolic acid can inhibit purine synthesis, suppress CD4^+^ T cell proliferation, and alter its phenotype, as well as activate the CD70 signaling pathway. Consequently, mycophenolic acid can reduce the production of the inflammatory cytokine IL-17 and inflammatory factors IFN-γ and TNF-α, decrease cellular oxygen consumption and glycolytic activity, and enhance the activity and function of Tregs (104).

Previous studies have revealed changes in the mitochondrial membrane potential and increased ROS in the lymphocytes of SLE patients (105). Treatment with N-acetylcysteine (NAC) can alleviate this phenomenon by reducing mTOR activity, thus increasing T cell mitochondrial membrane potential and cell apoptosis, as well as inducing the expression of nTregs in SLE patients (106). The above studies suggest that SLE is not only a typical autoimmune disease but also closely associated with immune metabolic abnormalities. Changes in immune metabolism play a crucial role in immune dysregulation, inflammatory responses, and the clinical manifestations of SLE.

### 4.5 Rheumatoid arthritis

Rheumatoid arthritis (RA) is a chronic autoimmune disease characterized by joint destruction and synovial inflammation, with severe cases potentially leading to disability (107). The development of RA is closely linked to changes in immune metabolism, particularly the metabolic reprogramming of Tregs, which plays a crucial role in the immune imbalance seen in RA. Alterations in metabolic pathways not only affect the quantity and function of nTregs but also have the potential to modulate the metabolic environment, thereby influencing the activity of immune cells and exacerbating inflammation and joint damage in RA (108).

Studies have shown that nutritional elements such as magnesium (Mg) play an important role in regulating immune metabolism in RA. Magnesium can reduce the production of pro-inflammatory cytokines such as TNFα, IL-1β, and IL-6, and exert anti-inflammatory effects by inhibiting NF-κB activation (109). A high-magnesium diet alters the metabolic environment of the gut microbiota, promotes the differentiation of Tregs, and increases the production of anti-inflammatory cytokine IL-10. This, in turn, reduces the differentiation of pathogenic Th17 cells, alleviates inflammation, and lessens the clinical symptoms of RA (110, 111). TP63 is highly expressed in Th17 cells; however, its expression is lower in nTregs because of its inhibition of Foxp3 (112). Methotrexate (MTX), which is a folate antagonist, is a standard first-line treatment for RA. It inhibits TP63 and Th17 cells while restoring the inhibitory function of nTregs, thereby improving RA (113).

TCR activation induces interleukin-2-inducible T cell kinase (ITK), which regulates the development of CD4^+^ T cell subtypes (114). Elevated levels of ITK have been observed in RA patients (115). The inhibitor of ITK known as dihydroartemisinin (DHA) acts by inhibiting the PI3K-Akt-mTOR signaling pathway. This mechanism results in a reduction in Th17 cells and an increase in nTregs, thus restoring the balance between the two cell types. As a result, the progression of RA is impeded (115, 116). Low-molecular-mass polypeptide (LMP7) is an immuno- proteasome subunit that can amplify inflammatory T cells and cytokines. It is highly expressed in patients with RA (117). Research indicates that the LMP7 inhibitor PR-957 can reduce the differentiation of Th1 and Th17 cells and promote the production of nTregs, thus alleviating RA (118). Smad7 is a key negative regulator of the TGF-β signaling pathway; specifically, it inhibits the TGF-β and NF-κB pathways, limits RA development, and maintains the balance between Th17 cells and Tregs (119). DNA methylation is a chemical modification that alters gene expression and is capable of changing chromosome structure and DNA stability, among other effects (120). Research has shown that DNA methylation of Smad7 in patients with RA can decrease its expression levels, thus leading to joint inflammation and damage and promoting Th17 cell differentiation while reducing nTregs differentiation (121).

The overexpression of Smad7 can inhibit the activation of NF-κB, block IL-1R and TNFR signaling, reduce proinflammatory cytokine levels and anti- apoptotic signal activity, thereby restoring the Th17/Treg balance and effectively alleviating RA to some extent (122). CD4^+^ T cells in the synovium of RA patients express L-type amino acid transporter 1 (LAT1), which is a potential diagnostic and therapeutic target for emerging diseases such as cancer (123, 124). The loss of LAT1 can inhibit Th17 cell differentiation, reduce the number of proinflammatory cells such as IFN-γ^+^ CD4^+^ T cells and TNF-α^+^ CD4^+^ T cells, maintain the quantity and suppressive capacity of nTregs, and significantly alleviate arthritis (123). LAT1 loss also downregulates AKT expression, thus inhibiting the PI3K-AKT-mTOR pathway, which is crucial for maintaining the Th17/Treg balance. Research has demonstrated that the LAT1 inhibitor JPH203 successfully alleviates arthritis severity in individuals with RA (125).

### 4.6 Multiple sclerosis

Multiple sclerosis (MS) is an autoimmune disease characterized by immune cell infiltration and abnormal immune responses in the central nervous system (CNS) (126). Currently, the experimental autoimmune encephalomyelitis (EAE) animal model is predominantly used in MS research (127). Th17 cells and Tregs are key participants in coordinating the immune response in MS, and their balance largely determines the autoimmune response in the central nervous system and the course of the disease. In MS patients, pro-inflammatory cytokines such as IL-1β, IL-6, TGF-β, and IL-23 are elevated. These cytokines not only affect the differentiation of nTregs but also influence their function through metabolic pathways such as glycolysis and lipid metabolism.

In metabolic regulation, Tregs rely on fatty acid oxidation (FAO) to maintain their suppressive function, while Th17 cells depend more on glycolysis. Therefore, changes in metabolic states, such as alterations in fatty acid and glucose metabolism, can directly affect Tregs’ functionality and promote the generation of pathogenic Th17 cells (128, 129). IL-23 is a crucial driving factor for the pathogenic subset of Th17 cells, thus leading to the development of EAE in mice, whereas nonpathogenic Th17 cells do not contribute to EAE development (130). nTregs in EAE mice are severely depleted and functionally impaired (131). The inhibitory capacity of nTregs in EAE is limited; although they can suppress the production of effector T cells and IFN-γ in the central nervous system, they cannot inhibit the production of IL-17 (132). Currently, the use of 2-DG to inhibit glycolysis, the loss of ACC1 and LDHA, and the use of statin drugs are essential pathways to reduce the differentiation of Th1 and Th17 cells, promote nTregs activation, and improve EAE (20, 33, 133). CTLA-4 can inhibit T cell activation and expansion, thus preventing the occurrence and development of inflammation (134).

In EAE mice, the lack of CTLA-4 in nTregs affects their inhibitory function, and supplementation with IL-10 compensates for the partial loss of CTLA-4, which partially restores the inhibitory function of nTregs (135). Treg-specific inducible PD-1 deficiency can also increase the levels of nTregs in the central nervous system and alleviate the severity of EAE (136). Additionally, the immunoreceptor tyrosine-based inhibitory motif (TIGIT) enhances the ability of Tregs to inhibit Th1 and Th17 cells by promoting the production of IL-10 (137). TGF-β signal transduction affects the differentiation of Tregs and Th17 cells by inducing Smad3 phosphorylation to induce Foxp3 and inhibit IL-6 and IL-23, thereby suppressing the differentiation of Th17 cells and reducing the severity of EAE (138).

An imbalance of Th17/Treg cells in MS also leads to changes in lipid profiles. Unsaturated fatty acids such as oleic acid induce the expression of IL-10 and adiponectin to activate AMPK and inhibit the production of IL-2 and IFN-γ (139). The PPAR family of lipid receptors regulates the differentiation of Th17 cells and Tregs by modulating IL-2 (140). Long-chain fatty acids (LCFAs) and short-chain fatty acids (SCFAs) have opposing effects on T cell differentiation. Specifically, LCFAs promote the differentiation of Th17 cells, thus contributing to the production of IFN-γ and IL-17, whereas SCFAs induce the proliferation of Tregs, thus increasing the expression of FOXP3 (141). Insulin-like growth factor (IGF) is a key regulatory factor in the balance of Th17/Treg cells, primarily by activating AKT and mTOR to suppress the developmental program of early-stage nTregs (142). IGF1R signal transduction promotes glycolysis and activates the PI3K-AKT-mTOR pathway, thereby promoting the differentiation of pathogenic Th17 cells and limiting the activity of nTregs. Therefore, the lack of IGF1R in EAE can reduce pathogenic Th17 cells, thereby alleviating the severity of EAE.

In terms of amino acid metabolism, glutamine uptake increases during Th17 cell activation, thus enhancing the differentiation of Th17 cells and making them pathogenic. The activation of mTOR promotes their pathogenicity during EAE (143). When the glutamine supply is insufficient, initial CD4^+^ T cells begin to differentiate into pTregs, which results in a corresponding decrease in the production of Th17 cells, thereby mitigating the severity of EAE (144). Additionally, prolonged inflammation in MS leads to changes in the phenotype and function of nTregs, thus resulting in the production of new pathogenic Treg subsets (Th1-like Tregs). This type of Treg, via the activation of the PI3K-AKT-mTOR pathway, secretes IFN-γ and can also play a role in promoting disease (145).

### 4.7 Arteriosclerosis

Arteriosclerosis (AS) is a chronic inflammatory disease characterized by the formation of atherosclerotic plaques in arterial walls, thus leading to reduced blood flow or direct blockage; moreover, it is a major cause of cardiovascular diseases (146). During AS, the imbalance between pathogenic Th1 and Th17 cells and protective Th2 and pTregs is a major factor in the occurrence and development of the disease (147). Research indicates that IFNγ secreted by Th1 cells can promote the deterioration of atherosclerotic plaques through various mechanisms, including the promotion of the recruitment of white blood cells and the formation of foam cells, as well as the inhibition of the proliferation of vascular smooth muscle cells and the production of collagen; in contrast, pTregs can effectively prevent the occurrence and development of AS in mice (148). Therefore, maintaining the balance between Th1 cells and pTregs helps to alleviate the progression of arteriosclerosis.

AS leads to a decrease in the number and impaired function of pTregs, as well as a decrease in IL-10 and TGF-β (149). Studies have shown that IL-10 secreted by pTregs can reduce the expression of the inflammatory factor IFN-γ, thus inhibiting AS; furthermore, the overexpression of TGF-β can also have the same effect (150). The microenvironment under AS promotes glycolysis, and Tregs are well suited to this low-glucose, high-lactate environment. However, although this environment promotes glycolysis, the expression of Foxp3, the key transcription factor of Tregs, is affected, leading to impaired proliferation and function of Tregs that should otherwise adapt to this environment, thereby contributing to pathogenesis (25).

Hypoxia is a key driving factor for the formation of atherosclerotic plaques; however, its regulation of Tregs is controversial (151). Some studies have suggested that hypoxia can increase the expression of Foxp3, thus promoting the differentiation and inhibitory functions of Tregs; however, other studies have indicated that hypoxia induces the ubiquitination and degradation of Foxp3 and, in the absence of HIF-1α, contributes to the differentiation of Tregs and the inhibition of the activation of Th17 cells (152). Therefore, the mechanism by which Tregs act on AS in a hypoxic environment still requires further exploration. As self-antigens in AS are presented to T cells by major histocompatibility complex class II (MHC II) molecules on antigen-presenting cells (APCs) and DCs are the predominant APCs, DCs play an indispensable role in atherosclerotic lesions. pTregs can inhibit DCs *via* coinhibitory molecules such as CTLA-4, thus disrupting T cell activation and reducing AS (153).

Macrophages can secrete proinflammatory cytokines, accelerating the formation of atherosclerotic plaques. Elevated plasma cholesterol levels also lead to AS, affecting the differentiation and function of pTregs. The accumulation of cholesterol alters the phenotype of pTregs, transforming them into proinflammatory Th1-like Tregs. These Tregs secrete the proinflammatory cytokine IFN-γ, activating the IFN-γ/IL-27-mediated STAT1 signaling pathway. As a result, Tregs, which originally have immunosuppressive functions, partially lose their suppressive activity, rendering them ineffective in controlling arterial inflammation and thereby accelerating the progression of atherosclerosis (154). pTregs can inhibit the secretion of inflammatory factors by macrophages, downregulate scavenger receptors on macrophages, reduce cholesterol absorption, and inhibit the formation of foam cells, which are required for AS development (155). As hydroxymethylglutaryl-coenzyme A (HMG-CoA) reductase inhibitors, statins can induce the accumulation of pTregs in atherosclerotic plaques and have a protective effect (156). In addition, the use of apolipoprotein A1 (ApoA1) can increase the expression of the cholesterol efflux transporter ABCA1 in pTregs, restore cholesterol levels, prevent the transformation of pTregs into follicular helper T (Tfh) cells, and thereby improve AS (157).

It has been reported that disturbances in amino acid metabolism also affect the development of AS (158). Some studies have suggested that supplementation with high arginine can activate CD4^+^ T cells and alleviate atherosclerotic plaques via the inhibitory ability of pTregs (159). Furthermore, IDO can induce the differentiation of pTregs and reduce the extent of AS lesions (160).

### 4.8 COVID-19

At the end of 2019, cases of pneumonia of unknown origin first appeared in Wuhan, China, and later led to a widespread outbreak. This sudden pneumonia was later diagnosed as coronavirus disease 2019 (COVID-19) caused by the novel coronavirus severe acute respiratory syndrome coronavirus 2 (SARS-CoV-2) (161). To date, more than one hundred million people have been affected by COVID-19, with a significantly higher probability of infection for elderly individuals, individuals with comorbidities, and cancer patients (162). The onset of COVID-19 is accompanied by inflammation and metabolic disturbances (163). COVID-19 infection can activate Th17 cells and suppress Tregs, thus disrupting the balance of Th17/Treg cells and leading to mitochondrial dysfunction (164). Compared to healthy individuals, COVID-19 patients show increased levels of inflammatory factors IL-6 and TNF-α due to the activation of Th17 cells, along with elevated expression of PD-L1 in CD8^+^ T cells and iTregs, accompanied by disturbances in tryptophan, amino acid, and glucose-lipid metabolism (165).

Research on the immunometabolism of Tregs in COVID-19 is relatively limited; therefore, we have summarized some of the existing studies. Several metabolic pathways pose a risk for increased severity of COVID-19. Studies have shown that patients with metabolic diseases such as diabetes and obesity have an increased mortality rate, which is associated with increased glycolysis and mitochondrial dysfunction in the immune cells of COVID-19 patients: Elevated blood glucose levels directly promote SARS-CoV-2 replication and pro-inflammatory cytokine expression. Glycolytic flux is essential for viral replication, and SARS- CoV-2-induced mtROS production stabilizes HIF-1α, thereby influencing glycolysis and cytokine expression (166). In patients with COVID-19 and diabetes, the levels of pro-inflammatory cytokines such as IL-6 are significantly elevated (167). Furthermore, COVID-19 alters tryptophan metabolism, in which IFN-γ-regulated IDO catalyzes the conversion of tryptophan to kynurenine, thus leading to reduced tryptophan and weakened antitumor capabilities (165). Changes in these metabolic pathways involve the regulation of Th17 cells and Tregs. Currently, treatments targeting TNF-α and IFN-γ are effective at reducing mortality in COVID-19-infected mice (168).

### 4.9 HIV

HIV/AIDS is a chronic disease characterized by immune activation and chronic inflammation. HIV infection can lead to immune metabolic imbalance, with CD4^+^ T cells being its primary target (169). The proliferation, differentiation, maintenance, and migration of iTregs to inflammatory sites require glycolysis. Therefore, in HIV patients, an environment with high glycolysis is often observed, thus leading to an increased number of iTregs (170). During HIV infection, HIV replication directly or by activating innate immune cells induces an increase in the number of iTregs in peripheral blood and mucosal tissues. At the same time, Th17 cells are rapidly depleted, disrupting the Th17/Treg balance. This imbalance leads to systemic immune dysfunction and triggers chronic inflammation (171).

HIV is often co-infected with HBV, and HIV or HIV gp120 can bind to co-receptors such as CCR5 and CXCR4, activating the AKT and ERK signaling pathways, which upregulate HIF-1α in hepatocytes and hepatic stellate cells. This upregulation increases HBV-induced TGF-β1 and pro-fibrotic gene expression, promoting liver fibrosis. Additionally, TGF-β1 can upregulate HIF-1α expression via the SMAD signaling pathway, forming a positive feedback loop that exacerbates the progression of liver fibrosis (172). Under hypoxic conditions, HIF-1α promotes glycolysis and *de novo* fatty acid synthesis while inhibiting OXPHOS and carnitine palmitoyl- transferase 1A (CPT1A), thereby reducing FAO. HIF-1α promotes Th17 cell differentiation, but its impact on the increase or decrease of Tregs remains controversial (173).

HIV infection results in lower GLUT1 levels in Tregs than in Th17 cells. In contrast, HIV infection increases the number of pathogenic Th17 cells, which are more glycolytic than nonpathogenic Th17 cells. Moreover, via PI3K/AKT-mediated inhibition of mTOR, TGF-β1 promotes iTreg differentiation, reduces the expression of GLUT1 and HK2 in iTregs, inhibits glycolysis, and promotes FAO (174). Although iTregs play a protective role by suppressing immune activation, studies have indicated that iTregs primarily trigger specific immunity in HIV, which is harmful. This effect could be observed through the imbalance between Th17 cells and Tregs (171).

In terms of lipid metabolism, HIV patients exhibit decreased expression of PPAR-γ and pro-lipid genes, which are necessary for lipid synthesis and storage. This suggests that FAO is weakened during HIV infection (175) and that weakened FAO may reciprocally inhibit iTregs production (176). The purine pathway involves various high-energy phosphorylated compounds, such as ATP (177), and it functions via the ectonucleotidases CD39 and CD73, thus generating immunosuppressive adenosine, which binds to G protein-coupled purinergic receptor 1 (P1) (178). P1-adenosine signaling in effector T cells promotes iTregs differentiation and ensures their inhibitory activity (179). An increased proportion of CD39 Tregs is a marker of HIV progression. This can weaken the immunosuppressive activity of HIV-specific CD8^+^ T cells and the ability of CD8^+^ T cells to inhibit HIV replication via cAMP (180). During HIV infection, an increased proportion of iTregs is associated with Tregs IDO induction and activity, as well as CTLA-4 expression. CTLA-4 expressed by iTregs can interact with B7 expressed by DCs, thus promoting IDO expression, which leads to an increase in the proportion of iTregs and a decrease in the proportion of Th17 cells (181).

Currently, antiretroviral therapy (ART) for HIV has transformed it from an almost fatal disease to a manageable chronic condition; however, the complete eradication of HIV has not been achieved (182). A consensus has not been reached concerning the impact of ART on the balance between Tregs and Th17 cells. Some studies have suggested that ART can decrease the proportion of iTregs in patients, whereas others have argued that ART cannot restore the proportion of iTregs to normal levels in treated patients. Similarly, there is controversy regarding changes in Th17 cells (183). The main reason for these disparate results may involve differences in ART duration, methods, and Th17/Treg identification methods (184).

## 5 Discussion

Immunometabolism has become a popular research area in recent years. Crosstalk between immune cells and metabolism is a dynamic process involving the activation, inflammation, and resolution of immune cells. This entire process involves numerous metabolic mechanisms, which can be summarized as changes in the phenotype, differentiation, and function of immune cells under the influence of the microenvironment, thus leading to the reprogramming of various metabolic pathways. Therefore, mutual coordination between the functions of immune cells and metabolic activities is crucial for maintaining overall immune metabolic environment homeostasis.

As negative regulators of immune metabolism, Tregs play a role in immune tolerance and in maintaining immune homeostasis. Tregs participate in various metabolic pathways, including glycolysis, lipid metabolism, and amino acid metabolism; moreover, they play different roles in these pathways. The mTOR pathway and glycolysis support Treg development, proliferation, and migration, while OXPHOS enhances their suppressive abilities.

Tregs play dual roles in different metabolic diseases, and the maintenance of the normal differentiation and function of Tregs is the key to maintaining immune metabolic balance. The absorption and discontinuation of nutrients and elements, as well as targeted drug therapy, are important methods for regulating the differentiation and function of Tregs and for restoring metabolic pathways. Different hormones and drugs can have markedly different effects on the proliferative capacity and inhibitory function of Tregs. Insulin and leptin can inhibit the proliferation and function of Tregs, whereas glucocorticoids, rapamycin, metformin, and pioglitazone can maintain and promote the differentiation and function of Tregs. Therefore, Treg-based immuno- therapy contributes to a deeper understanding of Treg differentiation, migration, and function, as well as innovation and optimization of treatment methods.

With the advancement of single-cell sequencing technology in recent years, the related subgroups and cytokines of Tregs will be further revealed. According to various single-cell transcriptomic analyses, Tregs can adapt to different pathological environments and establish entirely new transcriptional characteristics.

In summary, Tregs are a unique immune-tolerant regulatory cell type that plays an indispensable role in maintaining immune system balance and acts as a crucial component of immune metabolism. Therefore, a better understanding of their mechanism of action through the key signaling pathways and metabolic pathways in which they participate is essential for developing Treg cell-based therapeutic drugs and for providing new perspectives for the development of the field of immunometabolism.
